# The Tree That Hides the Forest

**DOI:** 10.5334/jbsr.1539

**Published:** 2018-04-19

**Authors:** Sammy Tawk, Benoît Ghaye

**Affiliations:** 1 Cliniques Univeristaires Saint-Luc, BE

**Keywords:** Chest radiography, mediastinal lines, spondylitis, atypical presentation

**Figure 1:**
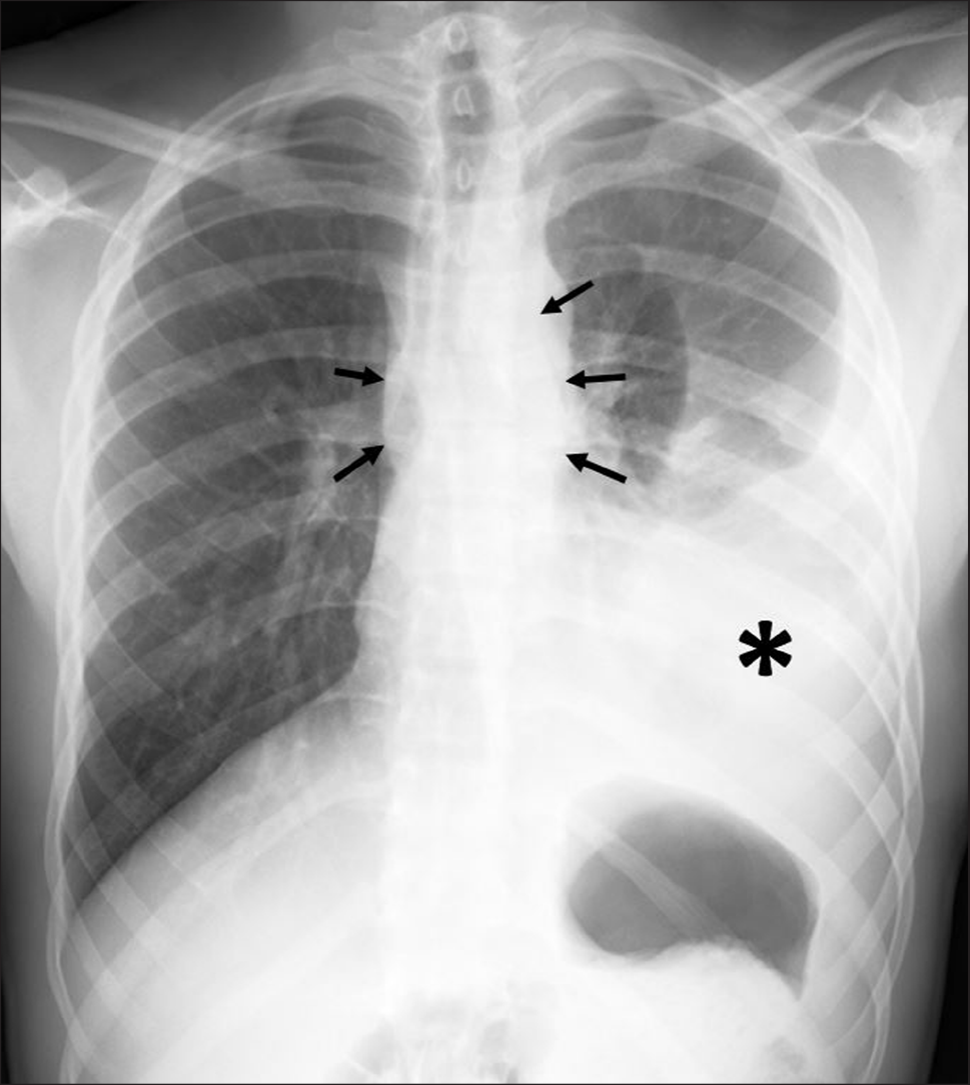
Postero-anterior (PA) chest radiograph showing large left pleural effusion (asterisk) and bilateral bulging of paraspinal lines (arrows).

A 22-year-old previously healthy male patient presented to the emergency department with a two-month history of fatigue, low-grade fever and progressive dyspnea. Chest radiography showed a large left pleural effusion with subtle bilateral enlargement of the superior mediastinum (Figure 1, arrows). Thoracentesis was performed for therapeutic and diagnostic purposes. Pleural fluid analysis was consistent with lymphocyte-rich exudate.

**Figure 2:**
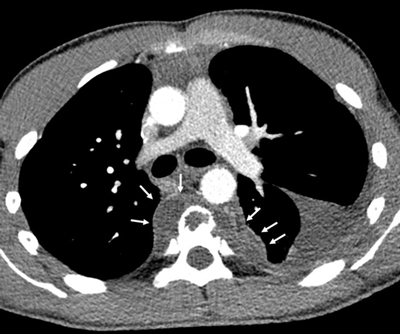
Contrast medium-enhanced axial CT scan of the chest (mediastinal window) at the pulmonary artery bifurcation level, showing the left pleural effusion (asterisk) as well as the pre-vertebral collections (arrows).

**Figure 3:**
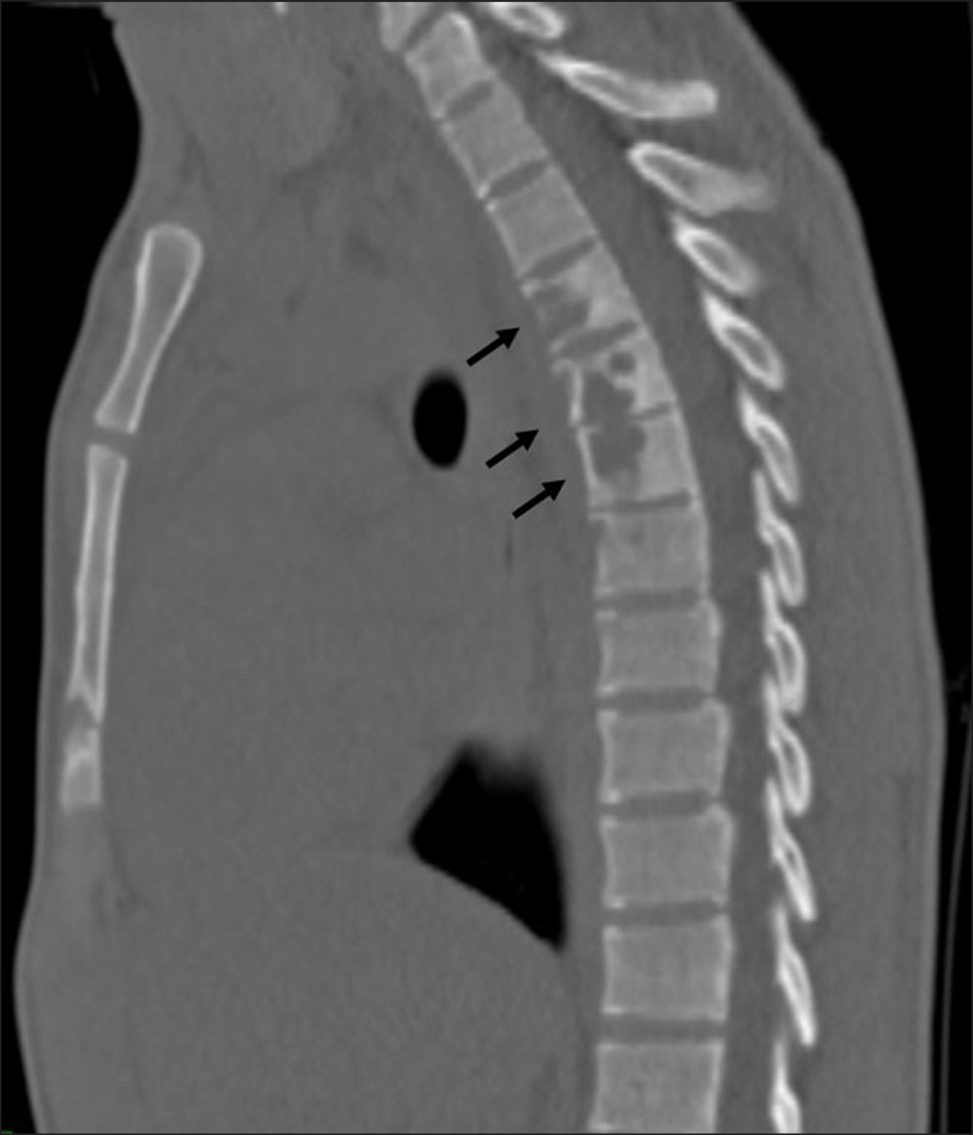
Reformatted sagittal CT scan image of the chest (bone window), showing extensive erosion involving the anterior aspect of three mid thoracic vertebral bodies (arrows). Note the subtle interrupted sclerotic margins as well as slight loss of height of the intervertebral disks in the affected segments.

Computed tomography (CT) of the chest revealed a pre-vertebral sub-ligamentous collection, extending bilaterally, and predominantly on the left (Figure 2, arrows) with an associated pleural effusion. Sagittal reformatted CT image in bone window showed extensive lysis of T4, T5, and T6 vertebral bodies, with some peripheral sclerosis and mild decrease in T5–T6 and T6–T7 disk height (Figure 3, arrows).

Imaging findings were consistent with tuberculous spondylodiscitis (confirmed by microbiological studies), an associated prevertebral collection and reactive left pleural effusion.

Tuberculous spondylodiscitis, also known as Pott’s disease, refers a to spinal infection caused by mycobacterium tuberculosis. It is characterized by an insidious onset and gradual progression of the disease compared to pyogenic spondylodiscitis. Although the final diagnosis can only be made by direct analysis of the infected tissue, constellation of radiological findings, clinical manifestations and blood tests results can suggest the diagnosis with high degree of confidence [1].
